# Professional quality of life of hospital nurses: A cross-sectional study

**DOI:** 10.1016/j.ijnsa.2026.100586

**Published:** 2026-06-04

**Authors:** Wendy H Oldenmenger, Aliene Otte-Paul, Arianne Stoppelenburg

**Affiliations:** a Erasmus MC Cancer Institute, University Medical Center Rotterdam, Department of Medical Oncology, Rotterdam, the Netherlands; b Department of Science, Franciscus Gasthuis & Vlietland, Rotterdam, the Netherlands; c Erasmus MC, University Medical Center Rotterdam, Department of Public Health, Programme Healthcare Quality and Sustainability, Rotterdam, the Netherlands

**Keywords:** Nursing, Perceived social support, Professional quality of life, Turnover intention, Hospital, Compassion fatigue, Burnout, Compassion satisfaction, Secondary traumatic stress

## Abstract

**Background:**

Health care systems are struggling to meet the growing demands for nursing care due to a shrinking workforce. The interplay between workforce shortages, professional quality of life, and insufficient support structures threatens not only nurses’ well-being but also the quality of care. Addressing these factors is crucial to ensure the resilience of the nursing workforce and the quality of patient care.

**Objective:**

To examine the levels of the three dimensions of Professional Quality of Life (ProQoL): compassion satisfaction (CS), burnout (BO) and secondary traumatic stress (STS) among hospital nurses in the Netherlands.

**Design:**

Cross-sectional study

**Setting:**

One academic hospital, four teaching hospitals and five general hospitals

**Participants:**

1017 nurses

**Methods:**

Quality of life of the nurses was assessed using the ProQoL-5 questionnaire. Besides, social support was measured using the Multidimensional Scale of Perceived Social Support. Additionally, we measured fear of making clinical errors on a 7-point Likert scale. Multivariable regression analysis was conducted to identify variables associated with the three subscales of ProQoL.

**Results:**

Average age was 32.1 years (SD 11.9), and respondents had on average 11.7 years’ experience (SD 10.9). The majority were registered nurses (83.8%). 69% reported a moderate level of CS, 31% reported a high level. No nurses scored high on BO (64% low, and 36% moderate). 72% of nurses reported low-level STS, and 27.4% reported a moderate level.

Multivariable analysis revealed that higher levels of CS were significantly associated with nurses with a specialized education, who considered working in health care in the future, older nurses, and nurses who perceived higher levels of social support from family, friends and significant others.

Lower levels of burnout were associated with nurses with more experience, with specialized education, who considered working in health care in the future, who had low fear of making a professional error, and nurses who perceived higher levels of social support from family or friends.

Lower levels of STS were significantly associated with male nurses, nurses who considered working in health care in the future, who had low fear of making a professional error, and nurses who perceived higher levels of social support from family or friends.

**Conclusions:**

Nurses in Dutch hospitals report low to moderate levels of BO and STS, and high levels of CS and social support. Social support is associated with lower levels of BO and STS, and higher levels of CS, and may serve as a protective factor.

Social media

To promote professional quality of life, hospitals need to create a healthy work culture and foster an engaged, and productive workforce

**Registration:**

Not registered

**What is already known**

• Growing complexity of healthcare needs increased psychosocial pressures on nurses.
• Social support improves the quality of life of nurses and the quality of care.

Alt-text: Unlabelled box dummy alt text

**What this paper adds**

• This study found that nurses working in Dutch hospitals experience high social support.
• The majority of nurses reported low levels of burnout and secondary traumatic stress.
• Most nurses reported moderate to high levels of compassion satisfaction.

Alt-text: Unlabelled box dummy alt text

## 1 Background

The nursing profession is widely recognized as both demanding and indispensable, with nurses forming the backbone of health systems worldwide. However, the growing complexity of healthcare needs, and medical treatments and technologies, coupled with ongoing workforce shortages, has intensified the psychosocial pressures nurses face. This puts them at a higher risk for burnout and compassion fatigue (Juraschek et al., 2019; European Commission, 2024). Burnout, involving emotional exhaustion, depersonalization and a lack of personal fulfillment at work, is common among nurses and linked to chronic workplace stress and insufficient organizational support (Maslach and Leiter, 2016; Dall'Ora et al., 2020). In parallel, Secondary traumatic stress emerges from the sustained emotional burden of caring for patients in distress, often resulting in reduced empathy and emotional withdrawal (Sinclair et al., 2017). In contrast, compassion satisfaction, which is the positive emotional reward derived from helping others, can serve as a protective factor, bolstering resilience and professional fulfilment (Stamm, 2010; Yesil and Polat, 2023).

The interplay between burnout, secondary traumatic stress and compassion satisfaction has significant implications for the well-being and quality of life of nurses. High levels of burnout and secondary traumatic stress are consistently associated with psychological distress, sleep disturbances and reduced job satisfaction. This has been found to result in increased absenteeism and turnover intentions (Gomez-Urquiza et al., 2017; Ruiz-Fernandez et al., 2020; Oldenmenger et al., 2025). These outcomes not only compromise the health and quality of life of nurses, but also have a negative effect on healthcare systems as a whole, undermining patient safety, continuity of care and overall engagement (McHugh et al., 2021; Hall et al., 2016). Conversely, nurses who experience greater compassion satisfaction report higher levels of engagement, lower rates of emotional exhaustion and an improved quality of life. This has a positive impact on patient outcomes and organizational performance (Sacco et al., 2015).

A possible influencing factor of nurses’ quality of life is perceived social support. Support from colleagues, friends and family members has been shown to reduce burnout and secondary traumatic stress, while enhancing compassion satisfaction and better health outcome (Spence Laschinger et al., 2012; Singh et al., 2024). Effective social support contributes to a sense of belonging, shared purpose, psychological safety and higher job satisfaction (Schug et al., 2021; Pergol-Metko et al., 2023). Social support functions as a protective factor against workplace stress and serves as a safety valve against occupational stress (Lawrence et al., 2007). Studies demonstrate that in the nursing profession, emotional and material support from colleagues and caregivers is of greater importance than support from friends and family (Maruyama and Morimoto, 1996). Importantly, social support improves the quality of life of nurses and, by extension, the quality of patient care and patient outcomes (Schug et al., 2021; Copanitsanou et al., 2017).

In addition to social support, clinical errors represent a relatively underexplored psychosocial factor affecting nurses’ professional quality of life. Beyond their direct impact on patients, clinical errors can impose a substantial emotional and psychological burden on healthcare professionals , who are often described as "second victims" (Wu, 2000). Although patient safety research has focused largely on error prevention and reporting, the fear of committing clinical errors has received comparatively little attention in safety culture assessments. Yet, this fear may provide important insights into workplace conditions and nurses’ psychological well-being (Boyer et al., 2024). Following a medical error, clinicians often experience intense guilt and fears related to their professional reputation, job security, licensure, future practice, and the patient involved (Delbanco and Bell, 2007). In the context of increasing concern regarding burnout and mental health among healthcare professionals, addressing fear of clinical errors is particularly relevant, as it has been identified as a significant risk factor for burnout (Boyer et al., 2024). Certain groups within the nursing workforce may be especially vulnerable to these stressors.

Nursing students, for instance, are at elevated risk of burnout and secondary traumatic stress during their clinical internships, especially during the increasing workforce crisis. This is because they need to adapt to new clinical environments and acquire new clinical skills, all while managing the additional burden of their education (Yi et al., 2024; Welch, 2023). High levels of secondary traumatic stress or burnout have been shown to increase the risk of nursing students leaving their education programs, exacerbating workforce attrition (Yi et al., 2024; Rudman et al., 2014).

These challenges are particularly acute in the context of the global nursing shortage, which has reached critical proportions in many countries, including the Netherlands (European Commission, 2024). Like many healthcare systems across Europe, the Dutch system is struggling to meet the growing demand for nursing care due to a shrinking workforce, which is being exacerbated by high rates of burnout and early career attrition (European Commission, 2024). The interplay between workforce shortages, professional quality of life, and insufficient support structures threatens not only nurses’ well-being but also the overall sustainability of healthcare delivery. Addressing these interconnected factors is crucial to ensure the resilience of the nursing workforce and the quality of patient care (Yu et al., 2025; European Commission, 2024; Chiminelli-Tomas et al., 2025).

Although international evidence underscores the importance of professional quality of life for nurses, there is limited empirical data from the Dutch hospital context. Due to the organizational structure of healthcare in the Netherlands, persistent workforce shortages, and relatively high expectations for patient safety and quality of care, context-specific research is necessary to inform targeted interventions and policy decisions.

Therefore, this cross-sectional study aimed to examine the levels of compassion satisfaction, burnout and secondary traumatic stress, as key dimensions of Professional Quality of life, among nurses working in hospitals in the Netherlands, and identify individual and work-related factors associated with each of these dimensions Based on the aim, we formulated two research questions:

1. What are the levels of compassion satisfaction, burnout, and secondary traumatic stress among hospital nurses in the Netherlands?
2. Which individual and work-related characteristics are associated with compassion satisfaction, burnout, and secondary traumatic stress among hospital nurses in the Netherlands?

## 2 Method

This is a descriptive cross-sectional study to explore the level of professional quality of life, and factors associated with ProQoL among hospital nurses. Reporting is in line with the Strengthening the Reporting of Observational Studies in Epidemiology (STROBE) checklist (von Elm et al., 2007).

### 2.1 Setting and procedure

Data were collected from December 2024 to May 2025 from one academic medical center, four teaching hospitals, and five general hospitals in the Rotterdam region of the Netherlands. The academic medical center had 1200 beds, and in the other hospitals the number of beds ranged between 600 and 1045 (four hospitals), and between 200 and 500 (five hospitals). Many different wards were included in the study. The specific combination of patient categories per ward differed between the hospitals.

Data were collected by bachelor nursing students (European Qualifications Framework Level 6) who selected this project as part of their final graduation research during the last five months of their clinical internship. These students received weekly education in research methods at the University of Applied Sciences and were supervised by a nursing researcher (PhD-level).

The survey was developed using LimeSurvey, a secure and user-friendly online survey platform, and was pilot tested to assess clarity and technical functionality prior to data collection. The students distributed a link to the survey, which included an introduction and consent form, to eligible registered nurses and nursing students on their wards using convenience sampling. A cover letter described the aim of the study and provided contact information for the research team. The survey remained open for a period of three weeks per ward. Due to the indirect distribution strategy, an accurate response could not be determined. To ensure an adequate sample size per ward, students were required to recruit a minimum of 20 registered nurses or nursing students.

### 2.2 Outcome measurements

Socio-demographic and work-related characteristics were collected, including sex, age, years of experience as a nurse, years in their current position, the type of hospital, department, and intention to leave their position.

Professional quality of life was measured using the ProQoL-5 questionnaire. ProQoL comprises three subscales; compassion satisfaction (CS), burnout (BO), and secondary traumatic stress (STS) (Stamm, 2010). The questionnaire is a 30-item self-report scale rated on a 5-point Likert scale (ranging from 1=’never’ to 5=’very often’), with higher scores on each subscale indicate higher SC, BO, and STS values. The three subscales each range from 10 to 50. The scores can be categorized in each of the subscales into ≤22= low; 23-41= moderate; and ≥42= high. The Cronbach’s alpha for the ProQoL-5 was 0.88 for compassion satisfaction, 0.75 for burnout, and 0.81 for secondary traumatic stress (Stamm, 2010). In our sample, the Cronbach’s alpha were 0.87, 0.68, and 0.82 respectively.

Perceived social support was measured using the Multidimensional Scale of Perceived Social Support (MSPSS). The MSPSS comprises three subscales: family, friends, and significant others. The questionnaire is a 12-item self-report scale rated on a 7-point Likert scale (ranging from 1= very strongly disagree to 7= very strongly agree). The mean scale score ranging from 1 to 2.9 could be considered as low support; a score of 3 to 5 could be considered moderate support; and a score from 5.1 to 7 could be considered high support (Zimet et al., 1988). In our sample, Cronbach’s alpha for the total score was 0.94. The subscales for family, friends, and significant others were 0.94, 0.94, and 0.93, respectively. To assess the fear of making clinical errors, we used the question ‘During your daily activities, how often are you afraid of making a professional error that could jeopardize patient safety?’ Responses were rated on a 7-point Likert scale. Respondents were categorized into two groups: those indicating frequent fear (‘once a week’, ‘a few times a week’, or ‘every day’) were considered to have ‘high fear’, while others were considered to have ‘ low fear’ (‘never’, ‘at least a few times a year’, ‘at least once a month’, or ‘a few times a month’) (Boyer et al., 2024).

### 2.3 Statistical analysis

Descriptive statistics were used to describe the demographics of the sample, including age, sex, years of experience as a nurse, working hours per week, intention to stay working in a hospital, and fear of making clinical errors. Continuous variables are presented as means and standard deviations (SD), and categorical variables as frequencies and percentages.

Levels of ProQoL and Perceived Social Support were described using means and standard deviations (SD) for the total sample. In addition, mean scores were calculated for the three ProQoL subscales: compassion satisfaction, burnout, and secondary traumatic stress, as described previously. Where relevant, descriptive analyses were also stratified for nursing students and registered nurses (Boyer et al., 2024).

Univariate associations between the three ProQoL subscales and the individual and work-related variables, were examined using Pearson correlation coefficients for independent variables at the interval level, and Spearman rank correlation coefficients for independent variables at categorical level. Variables showing a statistically significant univariate association (p < 0.05) with a ProQoL dimension were considered candidates for multivariable analysis.

Subsequently, separate multivariable linear regression analyses were conducted for compassion satisfaction, burnout, and secondary traumatic stress. All selected variables were entered simultaneously into the regression models using the backward method. Categorical variables were included as dummy variables, with appropriate reference categories defined a priori (e.g., registered nurses versus nursing students). Assumptions of linear regression, including normality of residuals, linearity, and absence of multicollinearity, were assessed and met for all models. Statistical analyses were performed using SPSS version 28, and a two-sided p-value < 0.05 was considered statistically significant.

### 2.4 Ethical approval and consent to participate

The study protocol was reviewed and approved by the Institutional Ethical Review Board of Erasmus MC (MEC2024-0466). The study was conducted in line with established ethical guidelines and regulations.

All participants received information about the nature and purpose of the study. By completing the survey, they provided informed consent. Participation was entirely voluntary, and individuals could withdraw at any time without facing any consequences.

## 3 Results

In total 1017 respondents filled out the questionnaire, 376 from the academic medical center, 541 from the teaching hospitals and 100 from the general hospitals. The included respondents were predominantly female (88%) and registered nurses (84%), with an average age of 32.1 years (range 17-69 years) and who had on average 11.7 years’ experience (Table 1, and N Supplementary table S1).

**Table 1:** Sociodemographic characteristics.

	Total N=1017 N (%)
Sex Male Female Non-binary Don’t want to say	113 (11.1) 895 (88.0) 4 (0.4) 5 (0.5)
Age (years, mean (sd)) ≤35 year >35 year missing	32.1 (11.9) 727 (71.5) 287 (28.2) 3 (0.3)
Nursing role Student nurse Registered nurse	164 (16.1) 853 (83.9)
Specialized education Yes No	406 (39.9) 611 (60.1)
Job experience in health care (years, mean (sd))	11.7 (10.9)
Job experience at the ward (years, mean (sd))	5.3 (7.2)
Working hours/ week (hours, mean (sd)) ≤28 hours >28 hours	29.8 (5.1) 348 (34.2) 669 (65.8)

### 3.1 Professional quality of life

The average compassion satisfaction score was 39.4 (SD 4.8) on a scale from 10 to 50. Most respondents (68.8%) reported a moderate level of compassion satisfaction, while 30.7% reported a high level. The mean burnout score was 21.3 (SD 4.4). Most respondents (64.4%) scored low on burnout, while 35.6% scored moderately. Notably, none of the respondents scored high on burnout. The average secondary traumatic stress score was 20.0 (SD 5.4). Seventy-two percent of respondents reported a low level of secondary traumatic stress, and 27.4% reported a moderate level (Table 2).

**Table 2:** Outcome measurements.

	Total N=1017 N (%)
Intention to stay working in hospital Yes No	928 (91.2) 89 (8.8)
Fear of making professional error High Low Missing	251 (24.8) 761 (75.2) 5 (0.5)
Perceived Social Support from family, mean (SD) Low support Moderate support High support Missing	5.8 (1.2) 42 (4.1) 178 (17.5) 751 (73.8) 46 (4.5)
Perceived Social Support from friends, mean (SD) Low support Moderate support High support Missing	5.8 (1.1) 25 (2.5) 196 (19.3) 749 (73.6) 47 (4.6)
Perceived Social Support from significant others, mean (SD) Low support Moderate support High support Missing	5.9 (1.2) 38 (3.7) 168 (16.5) 765 (75.2) 46 (4.5)
Perceived Social Support Total score, mean (SD) Low support Moderate support High support Missing	5.9 (1.0) 16 (1.6) 162 (15.9) 774 (76.1) 65 (6.4)
Compassion satisfaction, mean (SD) Low level Moderate level High level	39.4 (4.8) 5 (0.5) 700 (68.8) 312 (30.7)
Burnout, mean (SD) Low level Moderate level High level	21.3 (4.4) 655 (64.4) 362 (35.6) -
Secondary traumatic stress, mean (SD) Low level Moderate level High level	20.0 (5.4) 736 (72.4) 279 (27.4) 2 (0.2)

Student nurses scored statistically significantly higher than registered nurses on compassion satisfaction 40.3 (SD 4.8) vs. 39.2 (SD 4.7) (P < 0.001, Supplementary material Table 2).

### 3.2 Outcomes

The average score of perceived social support was 5.8 (from friend and family) and 5.9 (from significant others and total score). Most respondents reported to receive high support on all subscales (Table 2). We found no differences between student nurses and registered nurses (Supplementary material Table 2).

A quarter of the respondents indicated to be afraid of making a professional error that could jeopardize patient safety (Table 2). More student nurses than registered nurses were afraid to make a professional error (41.5% vs. 21.4%, P < 0.001; Supplementary material Table 2).

Over ninety percent of the respondents had the intention to stay working in the hospital. Significantly more student nurses than registered nurses had the intention to work in the hospital in the near future (95.7% vs. 90.4%, P < 0.05). The most important reasons for leaving health care were insufficient salary, excessive workload, physical strain and emotional stress caused by working conditions.

### 3.3 Determinants of compassion satisfaction

Social support from family, friends, significant others and the total score, together with age, nursing role, specialized education, years of experience in health care, the intention to stay working in the hospital and the fear of professional errors were significantly associated with the level of compassion satisfaction (Supplementary material table 3). These statistically significant variables were used as input for the multivariable regression analysis. This analysis revealed that seven variables explained 18% of the variance of compassion satisfaction. Student nurses reported higher levels of compassion satisfaction than registered nurses. Nurses with a specialized education, who considered working in health care in the future, and older respondents reported higher levels of compassion satisfaction. Additionally, respondents who perceived higher levels of social support from family, friends and significant others reported higher levels of compassion satisfaction (Table 3).

**Table 3:** Multivariable regression analysis of characteristics related to professional quality of life.

		B	95% CI	p-value
Compassion satisfaction*				
Nursing role	Student nurse Registered Nurse	1.80 ref	1.02, 2.58	<0.001
Have you completed further education?	No Yes	-.869 ref	-1.45, -0.29	0.003
Are you considering working in health care in the future?	Yes No	3.097 ref	2.14, 4.06	<0.001
Age		0.56	0.03, 0.08	<0.001
Social Support from Significant others		.27	-0.02, 0.55	0.065
Social Support from Family		.43	0.12, 0.72	0.005
Social Support from Friends		.76	0.45, 1.08	<0.001
Burnout⁎⁎				
Experience in health care (years)		-0.04	-0.07, -0.02	<0.001
Have you completed further education?	Yes No	0.51 ref	-0.03, 1.03	0.06
Are you considering working in health care in the future?	Yes No	-2.93 ref	-3.8, -2.06	<0.001
Fear of making professional error	High Low	1.76 ref	1.18, 2.34	<0.001
Social Support from Family		-0.66	-0.91, -0.41	<0.001
Social Support from Friends		-0.90	-1.17, -0.62	<0.001
Secondary traumatic stress⁎⁎⁎				
Sexe	Male Female	1.01 ref	0.20, 1.82	0.014
Are you considering working in health care in the future?	Yes No	-2.11 ref	-3.20, -1.02	<0.001
Fear of making professional error	High Low	3.31 ref	2.60, 4.03	<0.001
Social Support from Family		-0.59	-0.91, -0.27	<0.001
Social Support from Friends		-0.75	-1.09, -0.40	<0.001

⁎ R^2^=.18, adj R^2^=.18, F=30.52, p<0.001.

⁎⁎ R^2^=.26, adj R^2^=.26, F=57.17, p<0.001.

⁎⁎⁎ R^2^=.19, adj R^2^=.18, F=43.53, p<0.001.

### 3.4 Determinants of burnout

The multivariable regression analysis revealed that six variables explained 26% of the variance of burnout. Respondents with more experience in health care reported lower levels of burnout. Nurses with specialized education, who considered working in health care in the future, and who had low fear of making a professional error reported lower levels of burnout. Besides, respondents who perceived higher levels of social support from family or friends reported lower levels of burnout (Table 3).

### 3.5 Determinants of secondary traumatic stress

The multivariable regression analysis revealed that five variables explained 18% of the variance of secondary traumatic stress. Male nurses, nurses who considered working in health care in the future, and who had low fear of making a professional error reported lower levels of secondary traumatic stress. Furthermore, respondents who perceived higher levels of social support from family or friends reported lower levels of secondary traumatic stress (Table 3).

## 4 Discussion

The findings of this cross-sectional study among 1017 Dutch hospital nurses show that these nurses scored moderate to high on compassion satisfaction, 64% scored low on burnout, and 72% scored low on secondary traumatic stress. Besides, respondents scored high on perceived social support from family, friends and significant others. A quarter of the respondents were afraid of making a professional error that could jeopardize patient safety, and 91.2% had the intention to stay working in their hospital. Compassion satisfaction was higher in nurses with a specialized education, who had the intention to stay, were older, and who perceived higher levels of social support from family, friends and significant others. Burnout was lower in nurses with more experience, with specialized education, who had the intention to stay, who had low fear of making a professional error, and nurses who perceived higher levels of social support from family or friends. Secondary traumatic stress was lower in male nurses, nurses who had the intention to stay, who had low fear of making a professional error, and nurses who perceived higher levels of social support from family or friends.

In this study, our respondents scored on average 39.4 on compassion satisfaction. Compassion satisfaction scores for nurses in Texas were comparable (40.1) (Crabtree-Nelson et al., 2022), however other studies have reported lower levels of compassion satisfaction (30.7-36.1) (Xie et al., 2021; Yesil and Polat, 2023; Wei et al., 2024; Ruiz-Fernandez et al., 2020). On the other hand, in the current study, respondents scored low on burnout (21.3) and secondary traumatic stress (20.0). Nurses in Turkey scored this on the same level (Yesil and Polat, 2023), however, in other studies nurses scored on average in the moderate range of burnout (23.4-29.2) (Xie et al., 2021; Crabtree-Nelson et al., 2022; Wei et al., 2024; Yi et al., 2024) and secondary traumatic stress (25.2-27.4) (Xie et al., 2021; Wei et al., 2024; Yi et al., 2024). Xie et al. (2021) described in their meta-analysis that burnout and secondary traumatic stress increased gradually until 2019, their endpoint (Xie et al., 2021). It is questionable whether this is also the case in our region, nurses in the current study reported considerable lower levels of burnout and secondary traumatic stress (burnout 21.3 vs. 27.3, and STS 20.0 vs. 26.3) (Xie et al., 2021).

“Three-quarters of both registered nurses and student nurses reported receiving high support from family, friends and significant others. However, other studies showed lower levels of social support. For example, 31.6% of Iranian nurses (n=241) reported high levels of social support (Shojaei et al., 2019), and 46.7% of Polish nurses reported high levels of social support (Dziedzic et al., 2025). Although earlier studies have shown that social support from colleagues is more beneficial than support from family and friends (Pergol-Metko et al., 2023; Pasricha et al., 2020), our results showed the opposite. Only social support from family and from friends was significantly associated with the three dimensions of ProQoL, suggesting it is a protective factor for ProQoL.”

“Another factor significantly associated with burnout and secondary traumatic stress was fear of making a professional error. In this study, a quarter of respondents reported high fear of making an error: 41.5% of student nurses and 21.4% of registered nurses. Boyer et al. reported that 31.6% of French nurses (N = 2,819) and 36.9% of less experienced healthcare professionals (less than one year of experience) reported high fear of making an error, which is more aligned with our student nurses. Furthermore, similar to our study, Boyer et al. demonstrated significant associations between the fear of making errors and burnout. These findings underscore the need for active professional support to retain our younger and less experienced nurses in the workforce. To make this possible, we must create a safe working environment where nurses feel free to speak up and supported.”

### 4.1 Implications for practice

To promote professional quality of life, it is important to encourage organizations to create a healthy work culture and foster an engaged, inclusive, and productive workforce that provides high-quality care to patients (Sabesan et al., 2025). Studies have shown that there is a link between workplaces that encourage and support professional wellbeing and better patient outcomes and higher patient satisfaction (Guille and Sen, 2024). Based on the ASCO-COSA-ECO joint statement (Sabesan et al., 2025), four principles for Advancing Nursing Workforce Sustainability were defined (Table 4). The first principle is a more strategic one. During periods of workforce shortages, organizations may view nurses as merely bedside helpers, forgetting that they are professionals. In order to retain nurses, it is crucial to take them seriously, provide them with educational and training opportunities for further specialization, safe staffing and fair compensation. In the second principle, nursing teams' culture, shared decision-making and safety are important for two reasons. First, they help nurses perform well professionally. Second, they act as a safety net when there are difficult situations in private life. Nurses who feel heard and who are involved in changes at their workplace are more motivated to take on their responsibilities (Wan et al., 2018). In the third principle, nurses should be empowered to lead changes in workplace culture. In most countries, nurses are not used to taking the lead, not even over their own workplace, although they are capable of doing so. Having nursing role models is important because they can provide guidance and inspiration, helping to shape the future of the nursing profession (van der Cingel et al., 2021). Mentorship programs to support junior nurses, and peer support groups can decrease moral stress (Oldenmenger et al., 2024). Lastly, the fourth principle is about establishing systems for ongoing wellbeing assessment and accountability. It is important to focus on well-being during initial nursing education (Martin et al., 2026; Bakker et al., 2021). In clinical practice, it is important to involve junior nurses and nursing students in assessing of team cohesion and morale. They experience the work environment differently than management does. In addition, a standard recurring evaluation from outside the team is recommended to measure overall team performance and wellbeing (Maassen et al., 2024)(Table 4). Different professional societies started initiatives comparable to these principles, however, it is important that they are adapted to the different practice settings.

**Table 4:** Principles for advancing nursing workforce sustainability.

**Principle 1: Healthcare systems should commit to nurse wellbeing through strategic policies and governance** Organizations should embed nurse wellbeing into mission statements, strategic plans, and governance structures. This includes safe staffing, fair compensation, protected time for education and research, access to mentoring and wellbeing metrics.	**Principle 2: Promote inclusive, team-based nursing cultures** Multidisciplinary collaboration, psychological safety, and shared decision-making are essential for high-quality care and nurse satisfaction. Diverse, inclusive teams with shared purpose and mutual respect are key to sustainable care delivery.
**Principle 3: Empower nurses to lead change in workplace culture** Nurses should be central in designing interventions, policies, and innovations that improve wellbeing and their workflow. Recognize nurses as leaders and change agents.	**Principle 4: Establish systems for ongoing wellbeing assessment and accountability** Use internal and external mechanisms to monitor wellbeing. National and international nursing bodies and institutions should collaborate on benchmarking and transparent reporting. Embed wellbeing into nursing education and onboarding.

Based on: Sabesan S, Levit LA, Ceelen W, Crul M, Garrett-Mayer E, Kirkwood MK, Oldenmenger WH, Price R, Ryan M, Subbiah I, Dégi CL, Winer EP. Principles for advancing healthy workplace cultures: an ASCO-COSA-ECO joint statement. Lancet Oncol. 2025 Oct;26(10):1270-1273. doi: 10.1016/S1470-2045(25)00474-7.

Although our current study showed relatively low levels of burnout and secondary traumatic stress due to our implementation of the four principles of a healthy workplace, we still have a nursing shortage. This is mainly due to the fact that the profession is aging, and the increasing complexity of care. However, lower pay also leads nurses to leave the profession early. In order to reduce nurses' intentions to leave the profession, it is important to pay attention to their well-being in order to reduce their levels of burnout and secondary traumatic stress and enhance their compassion satisfaction (Oldenmenger et al., 2024; Ruiz-Fernandez et al., 2020; McHugh et al., 2021).

### 4.2 Strengths and limitations

The high number of respondents (1017 nurses) and the number of included hospitals are considered strengths of this study. This study has some limitations. Due to its a cross-sectional design, it was not possible to detect any causal relationships. Although we surveyed a large group of nurses from ten different hospitals, there may be selection bias, meaning this sample may not fully represent the Dutch hospital nurses. The nurses who participated in our study were very young. This is primarily due to the significant number of nursing students. However, this reflects daily practice in the Netherlands, where about a third of the nursing workforce consists of students working in hospitals. Additionally, registered nurses were also relatively young (34 years). It is possible that the student nurses encouraged their peers to participate in the survey. Furthermore, the survey was administered online, which may have resulted in a lower participation rates among older nurses. Nevertheless, young nurses are the future of the nursing workforce, so it is important to provide them with additional guidance to help them develop the resilience needed to stay in the nursing profession. Furthermore, fear of clinical error was assessed using a single, study-specific item. The cut-off applied to distinguish between ‘high’ and ‘low’ levels of fear was inherently arbitrary, which may have resulted in misclassification and an underestimation of fear prevalence. Furthermore, this dichotomization does not fully reflect the multidimensional and continuous nature of fear experiences among healthcare professionals. Nevertheless, we used the same methodology as in the original article (Boyer et al., 2024). Finally, our multivariable analysis revealed low variances (18%-26%). This indicates that much of the variance in ProQoL remains unexplained. Other factors may play a role here, and should be explored in future research.

## 5 Conclusion

Nurses working in Dutch hospitals report low to moderate levels of burnout and secondary traumatic stress, as well as high levels of compassion satisfaction and perceived social support. The findings further indicate that social support and nurses’ intention to continue working in healthcare are important factors, associated with lower burnout and secondary traumatic stress, as well as higher compassion satisfaction. These results underscore the pivotal role of supportive organizational environments in sustaining nurses’ ability to cope with the emotional demands of hospital care. Looking ahead, managers and policymakers should prioritize developing and implementing evidence-based strategies that enhance social support, ensure sustainable workloads, and protect nurses’ well-being over time. These strategies are essential for retaining the current nursing workforce and enhancing the long-term attractiveness of hospital nursing. Addressing these factors systematically and sustainably is crucial for mitigating the nursing shortage and ensuring continued access to high-quality, safe patient care.

## CRediT authorship contribution statement

**Wendy H Oldenmenger:** Writing – review & editing, Writing – original draft, Visualization, Supervision, Resources, Project administration, Methodology, Investigation, Formal analysis, Data curation, Conceptualization. **Aliene Otte-Paul:** Writing – review & editing, Resources, Conceptualization. **Arianne Stoppelenburg:** Writing – review & editing, Writing – original draft, Visualization, Supervision, Software, Resources, Project administration, Methodology, Data curation, Conceptualization.

## Declaration of competing interest

The authors declare that they have no known competing financial interests or personal relationships that could have appeared to influence the work reported in this paper.

## Appendix Supplementary materials

Image, application 1
