# Supplementary material for: Professional quality of life of hospital nurses: A cross-sectional study
Source: Int J Nurs Stud Adv. 2026 Jun 4;11:100586. doi: 10.1016/j.ijnsa.2026.100586 (PMC13264103; doi:10.1016/j.ijnsa.2026.100586)
Supplement: Supplementary file 1 [file mmc1.docx]

**Supplementary material table 1. Sociodemographic characteristics**

|  | **Student nurses**  **N=164**  **N (%)** | **Registered Nurses**  **N=853**  **N (%)** | **Total**  **N=1017**  **N (%)** |
| --- | --- | --- | --- |
| **Sex**  **Male**  **Female**  **Non-binary**  **Don’t want to say** | 20 (12.2)  142 (86.6)  1 (0.6)  1 (0.6) | 93 (10.9)  753 (88.3)  3 (0.4)  4 (0.5) | 113 (11.1)  895 (88.0)  4 (0.4)  5 (0.5) |
| **Age (years, mean (sd))**  **≤35 year**  **>35 year**  **missing** | 22.4 (4.3)  165 (100)  - | 34.0 (12.0)  563 (66.2)  287 (33.8)  3 | 32.1 (11.9)  727 (71.5)  287 (28.2)  3 (0.3) |
| **Specialized education**  **Yes**  **No** | 22 (13.4)  142 (86.6) | 384 (45)  469 (55) | 406 (39.9)  611 (60.1) |
| **Job experience in health care (years, mean (sd))** | 3.3 (2.0) | 13.3 (11.2) | 11.7 (10.9) |
| **Job experience at the ward**  **(years, mean (sd))** | 0.7 (0.7) | 6.2 (7.5) | 5.3 (7.2) |
| **Working hours/ week**  **hours, mean (sd))**  **≤28 hours**  **>28 hours** | 27.6 (5.2)  92 (56.1)  72 (43.9) | 30.2 (5.0)  256 (30)  597 (70) | 29.8 (5.1)  348 (34.2)  669 (65.8) |

**Supplementary material table 2. Outcome measurements**

|  | **Student nurses**  **N=164**  **N (%)** | **Registered Nurses**  **N=853**  **N (%)** | **Total**  **N=1017**  **N (%)** |
| --- | --- | --- | --- |
| **Intention to stay working in hospital**  **Yes**  **No** | 157 (95.7)*  7 (4.3) | 771 (90.4)*  82 (9.6) | 929 (91.2)  89 (8.8) |
| **Fear of making professional error**  **High**  **Low**  **Missing** | 68 (41.5)**  95 (57.9)  1 (0.6) | 183 (21.4)**  666 (78.1)  4 (0.5) | 251 (24.8)  761 (75.2)  5 (0.5) |
| **Compassion satisfaction**, mean (SD)  **Low level**  **Moderate level**  **High level** | 40.3 (4.8)*  -  96 (58.5)  68 (41.5) | 39.2 (4.7)*  5 (0.6)  604 (70.8)  244 (28.6) | 39.4 (4.8)  5 (0.5)  700 (68.8)  312 (30.7) |
| **Burnout,** mean (SD)  **Low level**  **Moderate level**  **High level** | 21.4 (4.4)  97 (59.1)  67 (40.9)  - | 21.3 (4.4)  558 (65.4)  295 (34.6)  - | 21.3 (4.4)  655 (64.4)  362 (35.6)  - |
| **Secondary traumatic stress,** mean (SD)  **Low level**  **Moderate level**  **High level** | 20.1 (5.5)  120 (73.2)  43 (26.2)  1 (0.6) | 20.0 (5.4)  616 (72.2)  236 (27.7)  1 (0.1) | 20.0 (5.4)  736 (72.4)  279 (27.4)  2 (0.2) |
| **Perceived Social Support from family**, mean (SD)  **Low support**  **Moderate support**  **High support**  **Missing** | 5.9 (1.3)  6 (3.7)  27 (16.5)  120 (73.2)  11 (6.7) | 5.8 (1.2)  36 (4.2)  151 (17.9)  640 (74.2)  35 (4.1) | 5.8 (1.2)  42 (4.1)  178 (17.5)  751 (73.8)  46 (4.5) |
| **Perceived Social Support from friends**, mean (SD)  **Low support**  **Moderate support**  **High support**  **Missing** | 5.8 (1.1)  3 (1.8)  33 (20.1)  116 (70.7)  12 (7.3) | 5.8 (1.2)  23 (2.7)  167 (19.4)  637 (73.9)  35 (4.1) | 5.8 (1.1)  25 (2.5)  196 (19.3)  749 (95.4)  47 (4.6) |
| **Perceived Social Support from significant others**, mean (SD)  **Low support**  **Moderate support**  **High support**  **Missing** | 5.9 (1.3)  8 (4.9)  21 (12.8)  124 (77.4)  11 (6.7) | 5.9 (1.2)  31 (3.6)  148 (17.2)  648 (75.2)  35 (4.1) | 5.9 (1.2)  38 (3.7)  168 (16.5)  765 (75.2)  46 (4.5) |
| **Perceived Social Support Total score**, mean (SD)  **Low support**  **Moderate support**  **High support**  **Missing** | 5.9 (1.0)  3 (1.8)  22 (13.4)  127 (77.4)  12 (7.3) | 5.9 (1.0)  17 (2.0)  143 (16.6)  667 (80.7)  35 (4.1) | 5.9 (1.0)  16 (1.6)  162 (15.9)  774 (76.1)  65 (6.4) |

*P<0.05; **P<0.001

**Supplementary material table 3. Univariate analysis**

|  | **Compassion satisfaction** | **Burnout** | **Secondary traumatic stress** |
| --- | --- | --- | --- |
| **Support family^p^** | .298** | -.372** | -.275** |
| **Support friends^p^** | .318** | -.394** | -.290** |
| **Support significant others^p^** | .270** | -.322** | -.200** |
| **SS_total^p^** | .343** | -.421** | -.296** |
| **Age^p^** | .065* | -.096** | - |
| **Sexe^s^** | - | - | .083 |
| **RN/ Student nurse^s^** | .082** | - | - |
| **Specialized education^s^** | -.092** | .112** | .063* |
| **Years experience health care^p^** | .097** | -.131** | - |
| **Years experience current ward^p^** | - | -.076* | - |
| **Intention to stay working in hospital^s^** | .159** | -.167** | -.102** |
| **Fear of errors^s^** | -.073* | .231** | .276** |
| **Type of hospital** | - | - | - |
| **Working hours per week** | - | - | - |

^s^Spearman correlation, as these variables are categorical variables

^p^Pearson correlation, as these are interval variables

*p<.05; **P<.001; - = not significant
